# Supplementary material for: Amoeboid-mesenchymal migration plasticity promotes invasion only in complex heterogeneous microenvironments
Source: Sci Rep. 2017 Aug 23;7:9237. doi: 10.1038/s41598-017-09300-3 (PMC5569097; doi:10.1038/s41598-017-09300-3)
Supplement: Supplementary file 1 — Supplementary information [file 41598_2017_9300_MOESM1_ESM.pdf]

Amoeboid-mesenchymal migration plasticity promotes invasion only in complex heterogeneous microenvironments

Katrin Talkenberger[1,*], Elisabetta Ada Cavalcanti-Adam[2,3], Anja Voss-Böhme[4,1], and Andreas Deutsch [1]

[1]Center for Information Services and High Performance Computing, Technische Universität Dresden, 01062 Dresden, Germany
[2]Department of Biophysical Chemistry, Institute of Physical Chemistry, Heidelberg University, 69120 Heidelberg, Germany
[3]Max Planck Institute for Medical Research, Department of Cellular Biophysics, 69120 Heidelberg, Germany
[4]Hochschule für Technik und Wirtschaft Dresden, Friedrich-List-Platz 1, 01069 Dresden
[*]Corresponding author: Katrin Talkenberger: katrin.boettger@tu-dresden.de

Supplementary Information

1 Model definition

We develop a probabilistic cellular automaton to study the impact of amoeboid-mesenchymal migration plasticity on tumor invasion. We consider a two-dimensional square lattice S with a fixed lattice spacing $\epsilon > 0$. Let N_r denote the nearest neighborhood of a lattice node $r \in S$. To each lattice node we assign a cell state value and an ECM resistance value. Let $\mathcal{E} = \{0, 1, 2\}$ be the set of possible cell states, where we interpret the cell state value 0 as unoccupied lattice node, 1 as an *amoeboid-like* (A) cell and the cell state value 2 as a *mesenchymal-like* (M) cell. Further, let $\mathcal{E}_{\text{ECM}} = [0, 1]$ be a countable set of ECM state values, with elements of $\mathcal{E}_{\text{ECM}}$ representing the ECM resistance. The state space of the whole lattice is specified by $(\mathcal{E} \times \mathcal{E}_{\text{ECM}})^S$.

Our cellular automaton is updated in discrete time $k \in \{i\tau | i \in \mathbb{N}\}$ with time step length $\tau > 0$. Let us consider a random variable $(\eta, \mu)(k) \in (\mathcal{E} \times \mathcal{E}_{\text{ECM}})^S$ describing the lattice configuration at time k. The time evolution of our cellular automaton model

$$(\eta, \mu)(k) \to (\eta, \mu)(k + 1)$$

is defined by the following update rule: At each discrete time step, select a node $r \in S$ at random. If the selected node is occupied, the cell at node r is updated by sequentially applying the following step:

(R1) *Phenotypic switch.*

- a cell changes its phenotype from A to M with rate $\alpha\mu(r)$, respectively from M to A with rate $\beta(1 - \mu(r))$.

(R2) *ECM degradation.*

- if the selected cell is of M-type, it locally degrades the ECM at site r with constant rate δ.

(R3) *Cell migration.*

- choose one node $r' \in N_r \setminus \{r\}$ with probability $p_G(r, r')$ (see below, equation (S.5)),
- if the neighboring node r' is empty, the cell moves from r to r' with rate $\lambda_A(\mu(r'))$ if the cell is of A-type, respectively with rate $\lambda_M(\mu(r'))$ for an M-cell,
- if the neighboring node r' is occupied, movement is aborted.

The above update rules are realized by subsequent application of a model-specific phenotypic switch rule

$\mathcal{R}^{\text{PS}}$, which describes the phenotypic switching (R1), an ECM degradation rule $\mathcal{R}^{\text{ECM}}$, which specifies (R2), and a local migration rule $\mathcal{M}_{rr'}$, which determines the cell movement (R3). During one discrete time step, the lattice configuration changes from $(\eta, \mu)(k)$ to $(\eta, \mu)(k+1)$ by,

$$(\eta, \mu)(k) := (\eta, \mu) \xrightarrow{\mathcal{R}^{\text{PS}}} (\eta', \mu) \xrightarrow{\mathcal{R}^{\text{ECM}}} (\eta', \mu') \xrightarrow{\mathcal{M}_{rr'}} (\eta'', \mu') =: (\eta, \mu)(k+1). \tag{S.1}$$

In our computer simulations, we set the time step length $\tau = 1$ [time unit] and the lattice spacing $\epsilon = 1$ [space unit]. For the implementation of the update rules, it is convenient to use probabilities instead of rates. For small enough time step length τ, we obtain phenotypic switch probabilities $p_\alpha := \alpha\tau$ and $p_\beta := \beta\tau$, a constant ECM degradation factor $c_\delta := \delta\tau$, and migration probabilities $p_{\lambda_A} := (\epsilon/\tau)\lambda_A$ and $p_{\lambda_M} := (\epsilon/\tau)\lambda_M$. In the following subsections, the precise model-specific update rules $\mathcal{R}^{\text{PS}}$, $\mathcal{R}^{\text{ECM}}$ and $\mathcal{M}_{rr'}$ are described in terms of transition probabilities.

1.1 Phenotypic switch dynamics

The local transition rule $\mathcal{R}^{\text{PS}}$ updates the state value of a node. In particular, during each automaton time step, the local phenotypic switch rule

$$\mathcal{R}^{\text{PS}} : \mathcal{E} \to \mathcal{E}, \; u \mapsto v \text{ with probability } W(u, v)$$

is applied, which updates the cell state value of a node $r \in S$. The transition probability W is specified by

$$W = (W(u,v))_{u,v \in \mathcal{E}} = \begin{array}{c} \\ {\scriptstyle u=0} \\ {\scriptstyle u=1} \\ {\scriptstyle u=2} \end{array} \begin{array}{c} \begin{array}{ccc} {\scriptstyle v=0} & {\scriptstyle v=1} & {\scriptstyle v=2} \end{array} \\ \begin{pmatrix} 1 & 0 & 0 \\ 0 & 1 - p_\alpha \mu(r) & p_\alpha \mu(r) \\ 0 & p_\beta(1 - \mu(r)) & 1 - p_\beta(1 - \mu(r)) \end{pmatrix} \end{array}. \tag{S.2}$$

Only such transitions occur where cells switch from A-type to M-type and vice versa. Please note that the switching process does not change the total number of cells but the ratio between A-type and M-type cells. The state value of the ECM resistance is not changed during the phenotypic switch step.

1.2 ECM degradation dynamics

According to our model rule (R2), the ECM is degraded with a constant factor $0 < c_\delta < 1$ only if an M-cell is present. The corresponding local ECM degradation rule is defined by

$$\mathcal{R}^{\text{ECM}} : \mathcal{E} \times \mathcal{E}_{\text{ECM}} \to \mathcal{E} \times \mathcal{E}_{\text{ECM}}, \; \mathcal{R}^{\text{ECM}}((u, \hat{u})) = \begin{cases} (u, (1 - c_\delta)\hat{u}) & \text{if } u = 2, \\ (u, \hat{u}) & \text{otherwise.} \end{cases} \tag{S.3}$$

1.3 Cell migration dynamics

The migration rule (R3) is that of a biased nearest-neighbor exclusion process [2, 4] of two cell types, where the direction of movement depends on an external gradient. Let us consider a family of *local transition rules* $(\mathcal{M}_{rr'})_{r,r' \in S}$, which is specified by

$$\mathcal{M}_{rr'} : \mathcal{E} \times \mathcal{E} \to \mathcal{E} \times \mathcal{E}, \; \mathcal{M}_{rr'}(u(r), v(r')) = \begin{cases} (0, u(r)) \text{ with probability } W_1(r, r') & \text{if } u(r) = 1, v(r') = 0 \\ (0, u(r)) \text{ with probability } W_2(r, r') & \text{if } u(r) = 2, v(r') = 0 \\ (u(r), v(r')) & \text{otherwise,} \end{cases} \tag{S.4}$$

where $(W_i(r, r'))_{r,r' \in S}$ are transition probabilities which have to satisfy

$$W_i : S \times S \to [0, 1] \text{ with } \sum_{r' \in S} W_i(r, r') = 1, \quad i = 1, 2.$$

The local transition rule $(M_{rr'})$ models the movement of A-cells and M-cells, respectively, by exchanging the state values of two nodes r and r', where a cell at node r only moves if the target node r' is empty (exclusion principle). The transition probabilities $W_1(r, r')$ and $W_2(r, r')$ specify the probabilities that an A-cell and an M-cell, respectively, move from r to r'. The movement probabilities depend on the presence of an external gradient G and on the state value of the ECM resistance at the target node r', as explained in the following.

The movement of A-cells and M-cells towards an external gradient is modeled with a function $p_G : S \times S \to [0,1]$ that depends on the movement direction $(r - r') \in N$ and the presence of a space-dependent external gradient $G \subset \mathbb{R}^d$,

$$p_G(r, r') = \frac{1}{Z(r)} e^{\kappa \langle (r-r'), G(r) \rangle}, \tag{S.5}$$

where $Z(r) = \sum_{i \in N} e^{\kappa \langle i, G(r) \rangle}$ is a normalization term and κ is the gradient sensitivity parameter, which controls the motility bias. The inner product $\langle \cdot, \cdot \rangle$ favors (or penalizes) the movement direction that tends to has the same (or inverse) direction of the gradient G. The jump probabilities p_{λ_A} and p_{λ_M} are modeled as functions $p_{\lambda_A}, p_{\lambda_M} : \mathcal{E}_{\text{ECM}} \to [0,1]$ that depend on the ECM resistance μ at the target jump site r'

$$\begin{aligned} p_{\lambda_A}(\mu(r')) &= c_A/(1 + \exp(15(\mu(r') - 0.5))), \\ p_{\lambda_M}(\mu(r')) &= c_M/(1 + \exp(15(\mu(r') - 0.5))), \end{aligned} \tag{S.6}$$

where c_A and c_M are constants, which are chosen such that the migration rate of A-cells is higher than that of M-cells for low to moderate ECM resistance. For $\mu = 0.5$, the slopes of the migration rate functions are steepest. We remark that the functional forms of the migration rate and switch functions are only one of many possibilities and are chosen for simplicity. The functions might be refined in response to future experimental results. Given the direction probability p_G and the jump probabilities p_{λ_A} and p_{λ_M}, the transition probabilities $W_1(r, r')$ and $W_2(r, r')$ are defined by

$$W_1(r, r') = \begin{cases} p_G(r, r') p_{\lambda_A}(\mu(r')) & \text{if } r' \in N_r \setminus \{r\}, \\ 1 - \sum\limits_{r' \in N_r \setminus \{r\}} p_G(r, r') p_{\lambda_A}(\mu(r')) & \text{if } r = r', \\ 0 & \text{otherwise}, \end{cases} \tag{S.7}$$

$$W_2(r, r') = \begin{cases} p_G(r, r') p_{\lambda_M}(\mu(r')) & \text{if } r' \in N_r \setminus \{r\}, \\ 1 - \sum\limits_{r' \in N_r \setminus \{r\}} p_G(r, r') p_{\lambda_M}(\mu(r')) & \text{if } r = r', \\ 0 & \text{otherwise}. \end{cases} \tag{S.8}$$

We remark that multi-species exclusion processes have been previously described and studied by Simpson et al. [3], where, however, the directional probabilities and the jump probability are chosen spatially constant.

2 Simulation study

2.1 Calculation details

Our model is simulated on a rectangular lattice $S = S_1 \times S_2$. At the initial time, A- and M-cells are placed at random along the left border of the lattice. In each simulation, we study the movement of either a switching population, that is when cells are allowed to change their phenotype $(\alpha, \beta > 0)$, or of a non-switching population $(\alpha = \beta = 0)$. We analyze the average distance which A- and M-cells move in the lattice for a given time. The *migration distance from the initial position of an individual cell* at position (r_1, r_2) is defined as the absolute distance the cell moved in the r_2-direction until the end of the simulation. In particular, if

$$n(r_1, r_2) = \begin{cases} 1 & \text{if } \eta(r_1, r_2) \neq 0 \\ 0 & \text{otherwise} \end{cases} \tag{S.9}$$

denotes the indicator of a cell presence at position (r_1, r_2) at the end of the simulation, we define

$$d = r_2 n(r_1, r_2) \tag{S.10}$$

for the traveled distance of a cell located at (r_1, r_2). The migration distance d is measured for each individual cell and the average is calculated for the entire cell population:

$$d_p = \frac{\sum\limits_{r_1=1}^{S_1} \sum\limits_{r_2=1}^{S_2} r_2 n(r_1, r_2)}{n}, \tag{S.11}$$

where n is the overall number of cells in the simulation, which is a conserved quantity. The maximum migration distance of the cell population is given by

$$d_{max} = \max_{r_2} \left(\sum_{r_1=1}^{S_1} r_2 n(r_1, r_2) \right). \tag{S.12}$$

Let $d_p^s = d_p^s(\alpha/\beta)$ denote the average migration distance d_p of the switching population depending on the phenotypic switch ratio $\alpha/\beta \in \{1/10, 2/10, \ldots, 1, 2, \ldots, 10\}$. Likewise, $d_p^n = d_p^n(\gamma)$ denotes the average migration distance d_p of the non-switching population depending on the fraction of M-cells $\gamma \in [0, 1]$. The difference between the switching population with maximum average migration distance d_p with respect to varied switch ratio α/β and the non-switching population with maximum average migration distance d_p with respect to varied fractions γ of M-cells is then given by

$$\Delta d_p = \max_{\alpha/\beta}\ d_p^s(\alpha/\beta) - \max_{\gamma}\ d_p^n(\gamma). \tag{S.13}$$

In the same way, the difference between the best switching population and the best non-switching population are considered with respect to d_{max}:

$$\Delta d_{max} = \max_{\alpha/\beta}\ d_{max}^s(\alpha/\beta) - \max_{\gamma}\ d_{max}^n(\gamma). \tag{S.14}$$

2.2 Monte Carlo step

A Monte Carlo step is the standard time unit in our system. In particular, let n be the total number of cells in our model. At each discrete time step, one cell is selected at random and updated according to our model rules. On average, each cell is updated once per n consecutive simulation time steps. The sequence of n time steps corresponds to one Monte Carlo step.

2.3 Different ECM resistance distributions

In our study, we have tested different heterogeneous ECM resistance distributions. In the main text, we present results of an heterogeneous ECM modeled by a sinusoidal function, see equation (2). Qualitatively similar results have been found for other functional representations of the ECM resistance distribution, such as

$$\mu(r_1, r_2) = 1 - \theta \exp\left(-\frac{1}{20S_1} r_1^2 - \frac{1}{100S_2} \left(r_2 - \frac{S_2}{4} \right)^2 \right) - (1-\theta)\xi \tag{S.15}$$

or

$$\mu(r_1, r_2) = \frac{\theta}{2} \cos\left(\frac{6\pi}{S_1} r_1 + \pi \right) + 1 - \frac{\theta}{2} - (1-\theta)\xi, \tag{S.16}$$

where $\xi \sim \mathcal{U}(0, 1)$ is a fixed uniformly distributed random variable and $\theta \in (0, 1)$ is a heterogeneity parameter. An illustration of the above two ECM resistance distributions is shown in Figure S1.

2.4 Equilibrium cell distribution

To explore whether our simulation results presented in the main text are sensitive to our choice of the initial cell density distribution, we repeated our simulation study for different initial cell distributions and under different ECM resistance structures. We find that independent of the initial cell distribution and independent of the underlying ECM resistance structure, an equilibrium distribution of A- and M-cells is reached after only a few Monte Carlo Steps.

2.5 Additional material: maximum migration distance and individual cell trajectories

Figures S3, S3, S5, S6 and S7 present additional material which fortifies our simulation results presented in the main text. Figure S5 shows the average and maximum migration distance of switching and non-switching cell populations under homogeneous, low (Figure S5(a)) and homogeneous, high (Figure S5(b)) ECM conditions. The figure also show the variance between simulations. Figures S3 and S4 illustrate possible cell trajectories of switching and non-switching populations under homogeneous, low (Figure S3) and homogeneous, high (Figure S4) ECM conditions.

Figure S6 shows the average and maximum migration distance of switching and non-switching cell populations under heterogeneous, highly structured ECM conditions with high (Figure S6(a)) and small (Figure S6(b)) migration rate ratio. The figure also show the variance between simulations. Figure S7 illustrates possible cell trajectories of switching and non-switching populations under heterogeneous, highly structured ECM conditions with large migration rate ratio (the corresponding trajectory figure for small migration rate ratio is shown in the main text.)

2.6 Sensitivity analysis of chemotactic responsiveness

Under homogeneous ECM conditions, the highest average migration distance d_p is observed for non-switching populations. This result is independent of the responsiveness towards the chemotactic gradient. In the main text, we have already shown data for homogeneous ECM with high chemotactic gradient responsiveness ($\kappa = 1$) in Figure 4. Here, in Figure S8, we show that our results also hold for low gradient responsiveness ($\kappa = 0.01$). In particular, Figure S8(a) shows the average migration spreading distance d_p of switching and non-switching populations under homogeneous, low ECM resistance, and Figure S8(b) under high ECM resistance conditions. The description of the results is analogous to that of Figure 4 in the main text.

Under heterogeneous, weakly structured ECM conditions, we observe that the non-switching behavior provides the highest average migration distance d_p. This result is independent of the chemotactic responsiveness, as illustrated in Figure S9. Figure S9(a) shows the average migration distance d_p of switching and non-switching populations under heterogeneous, weakly structured ECM, in the case that cells move undirected ($\kappa = 0$). We observe that the higher the portion of M-cells, the higher the average migration distance d_p of the non-switching population. Similarly, for the switching population, the higher the switch ratio α/β, that is cells are predominantly M-type, the higher the average migration distance d_p. As shown in Figure S9(b) and Figure S9(c), it becomes apparent that the higher the chemotactic responsiveness, the higher the average migration distanced_p of the switching and non-switching populations, where always the non-switching behavior shows maximum average migration distance d_p.

Under heterogeneous, highly structured ECM, we find that the switching behavior can lead to maximum average migration distance d_p. Figure S10 gives an overview of the observed dependency of the average migration distance d_p on the chemotactic responsiveness, the level of ECM heterogeneity and the migration rate ratio c_M/c_A. Figure S10(a) shows the parameter space which is explored to analyze the influence of the chemotactic responsiveness, ECM structure and migration rate ratio on the migration distance d_p. The phase diagrams in Figure S10(b)-S10(d) show the difference between the switching population with maximum average migration distance d_p with respect to varied switch ratio α/β and the non-switching population with maximum d_p with respect to varied M-cell fraction γ, depending on the ECM structure and the migration rate ratio, for different chemotactic responsiveness: no responsiveness (Figure S10(b)), low responsiveness (Figure S10(c)) and high responsiveness (Figure S10(d)). We observe that if the chemotactic responsiveness is increasing, the switching behavior is favorable if the ECM is highly structured and the migration rate ratio c_M/c_A sufficiently small.

3 Mean-field approximation of the probabilistic cellular automaton dynamics

In our study, we performed extensive numerical simulations of our cellular automaton model. In particular, the migration distance A- and M-cells move along the horizontal axis of a rectangular lattice for a given time is analyzed. In this section, we make a first attempt to develop an analytically accessible model to describe the migration behavior of switching (and non-switching) cell populations. From cellular automaton Rules (R1)-(R4), see section 1, we derive the corresponding partial differential equation, which describes the temporal changes of the A- and M-cell density profiles. We will proceed step by step. We derive a macroscopic description of the cell migration process first without and then with phenotypic switching. Subsequently, the ECM degradation process by a partial differential equation is approximated and the analysis of the complete macroscopic model is discussed.

3.1 Scaling limit of the cell migration process

In the following, we consider the automaton cell migration process without phenotypic switch. Based on the family $(\mathcal{M}_{rr'})_{r,r' \in S}$ of local migration rules, the cellular automaton migration process can be

formulated as discrete-time Markov chain on $\mathcal{E}^S$, with global transition probabilities

$$P(\zeta,\nu) := \begin{cases} \frac{1}{|S|}W_1(r,r') & \text{if } \zeta(r)=\nu(r')=1, \zeta(r')=\nu(r)=0 \\ \frac{1}{|S|}W_2(r,r') & \text{if } \zeta(r)=\nu(r')=2, \zeta(r')=\nu(r)=0 \\ \frac{1}{|S|}\sum_{r\in S} W_1(r,r)+W_2(r,r) & \text{if } \zeta=\nu \\ 0 & \text{else,} \end{cases} \tag{S.17}$$

for $\zeta,\nu \in \mathcal{E}^S$ and $r' \in N_r$. It is easy to verify that $\sum_{\nu\in\mathcal{E}^S} P(\zeta,\nu)=1$ holds. The change in occupation of the lattice node r during one automaton time step is described the discrete conservation equations

$$\begin{aligned} P(\eta(r,k+1)=u)-&P(\eta(r,k)=u) \\ &= \sum_{r'\in\mathcal{N}_r\setminus\{r\}} \frac{1}{|S|}W_u(r',r)P(\eta(r',k)=u,\eta(r,k)=0) \\ &\quad - \sum_{r'\in\mathcal{N}_r\setminus\{r\}} \frac{1}{|S|}W_u(r,r')P(\eta(r',k)=0,\eta(r,k)=u), \end{aligned} \tag{S.18}$$

for $u \in \{1,2\}$.

Let $\rho_a(r,k)$ and $\rho_m(r,k)$ denote the mean cell density of A-cells and M-cells, respectively, at node r at time k. Let further $\rho(r,k)=\rho_a(r,k)+\rho_m(r,k)$ denote the mean cell density of the total population. By invoking the mean-field assumption, equation (S.18) becomes

$$\begin{aligned} \rho_a(r,k+1)-\rho_a(r,k) &= \sum_{r'\in\mathcal{N}_r} \frac{1}{|S|}W_1(r',r)\rho_a(r',k)(1-\rho_a(r,k)-\rho_m(r,k)) \\ &\quad - \sum_{r'\in\mathcal{N}_r} \frac{1}{|S|}W_1(r,r')\rho_a(r,k)(1-\rho_a(r',k)-\rho_m(r',k)) \end{aligned} \tag{S.19}$$

and

$$\begin{aligned} \rho_m(r,k+1)-\rho_m(r,k) &= \sum_{r'\in\mathcal{N}_r} \frac{1}{|S|}W_1(r',r)\rho_m(r',k)(1-\rho_a(r,k)-\rho_m(r,k)) \\ &\quad - \sum_{r'\in\mathcal{N}_r} \frac{1}{|S|}W_1(r,r')\rho_m(r,k)(1-\rho_a(r',k)-\rho_m(r',k)), \end{aligned} \tag{S.20}$$

respectively. In the following, we present the derivation of a macroscopic description for the A-cell subpopulation in one dimension. The derivation for the M-cell subpopulation is carried out analogously. An extension to higher dimensions is straightforward.

In one dimension, the nearest neighborhood of a node r is $N_r=\{r-1,r,r+1\}$ and equation (S.19) can be written

$$\begin{aligned} &\rho_a(r,k+1)-\rho_a(r,k) \\ &\quad= \frac{1}{|S|}\Big(W_1(r-1,r)\rho_a(r-1,k)-W_1(r-1,r)\rho_a(r-1,k)a(r,k) \\ &\qquad - W_1(r-1,r)\rho_a(r-1,k)m(r,k)+W_1(r+1,r)\rho_a(r+1,k) \\ &\qquad - W_1(r+1,r)\rho_a(r+1,k)\rho_a(r,k)-W_1(r+1,r)\rho_a(r+1,k)\rho_m(r,k) \\ &\qquad + W_1(r,r-1)\rho_a(r,k)+W_1(r,r-1)\rho_a(r,k)\rho_a(r-1,k) \\ &\qquad - W_1(r,r-1)\rho_a(r,k)\rho_m(r-1,k)+W_1(r,r+1)\rho_a(r,k) \\ &\qquad + W_1(r,r+1)\rho_a(r,k)\rho_a(r+1,k)-W_1(r,r+1)\rho_a(r,k)\rho_m(r+1,k)\Big). \end{aligned} \tag{S.21}$$

For ease of notation, from now on we rewrite the jump probability and direction probability as

$$f(\mu_r) := p_{\lambda_A}(\mu(r,k)), \quad g(G_r) := p_G(r,r'). \tag{S.22}$$

Then the discrete conservation equation (S.21) reads

$$
\begin{aligned}
&\rho_a(r,k+1)-\rho_a(r,k)\\
&\quad=\frac{1}{|S|}\Big(f(\mu_r)(1-g(G_{r-1}))\rho_a(r-1,k)-f(\mu_r)(1-g(G_{r-1}))\rho_a(r-1,k)\rho_a(r,k)\\
&\qquad-f(\mu_r)(1-g(G_{r-1}))\rho_a(r-1,k)\rho_m(r,k)+f(\mu_r)g(G_{r+1})\rho_a(r+1,k)\\
&\qquad-f(\mu_r)g(G_{r+1})\rho_a(r+1,k)\rho_a(r,k)-f(\mu_r)g(G_{r+1})\rho_a(r+1,k)\rho_m(r,k)\\
&\qquad+f(\mu_{r-1})g(G_r)\rho_a(r,k)+f(\mu_{r-1})g(G_r)\rho_a(r,k)\rho_a(r-1,k)\\
&\qquad-f(\mu_{r-1})g(G_r)\rho_a(r,k)\rho_m(r-1,k)+f(\mu_{r+1})(1-g(G_r))\rho_a(r,k)\\
&\qquad+f(\mu_{r+1})(1-g(G_r))\rho_a(r,k)\rho_a(r+1,k)\\
&\qquad-f(\mu_{r+1})(1-g(G_r))\rho_a(r,k)\rho_m(r+1,k)\Big).
\end{aligned}
\tag{S.23}
$$

To derive the macroscopic behavior of our automaton migration dynamics, we scale the automaton lattice spacing and time step length, such that $x = r\epsilon \in \mathbb{R}$ and $t = \tau k|S| \in \mathbb{R}_+$, which gives

$$
\begin{aligned}
&\rho_a(x,t+\tau|S|)-\rho_a(x,t)\\
&\quad=\frac{1}{|S|}\Big(f(\mu_x)(1-g(G_{x-\epsilon}))\rho_a(x-\epsilon,t)-f(\mu_x)(1-g(G_{x-\epsilon}))\rho_a(x-\epsilon,t)\rho_a(x,t)\\
&\qquad-f(\mu_x)(1-g(G_{x-\epsilon}))\rho_a(x-\epsilon,t)m(x,t)+f(\mu_x)g(G_{x+\epsilon})\rho_a(x+\epsilon,t)\\
&\qquad-f(\mu_r)g(G_{x+\epsilon})\rho_a(x+\epsilon,t)\rho_a(x,t)-f(\mu_r)g(G_{x+\epsilon})\rho_a(x+\epsilon,t)\rho_m(x,t)\\
&\qquad+f(\mu_{x-\epsilon})g(G_x)\rho_a(x,t)+f(\mu_{x-\epsilon})g(G_x)\rho_a(x,t)\rho_a(x-\epsilon,t)\\
&\qquad-f(\mu_{x-\epsilon})g(G_x)\rho_a(x,t)\rho_m(x-\epsilon,t)+f(\mu_{x+\epsilon})(1-g(G_x))\rho_a(x,t)\\
&\qquad+f(\mu_{x+\epsilon})(1-g(G_x))\rho_a(x,t)\rho_a(x+\epsilon,t)\\
&\qquad-f(\mu_{x+\epsilon})(1-g(G_x))\rho_a(x,t)\rho_m(x+\epsilon,t)\Big).
\end{aligned}
\tag{S.24}
$$

Similar to the approach used in Chapter 3, the next step is to expand all terms on the right of equation (S.24) in a Taylor series around (x,t), keeping terms up to $\mathcal{O}(\epsilon^2)$. A Taylor expansion of $\rho_a(x\pm\epsilon,t)$, $f(\mu_{x\pm\epsilon})$ and $g(G_{x\pm\epsilon})$ yields

$$
\begin{aligned}
\rho_a(x\pm\epsilon,t)&=\rho_a\pm\epsilon\partial_x\rho_a+\frac{\epsilon^2}{2}\partial_{xx}\rho_a+\mathcal{O}(\epsilon^3)\\
f(\mu_{x\pm\epsilon})&=f(\mu)\pm\epsilon\partial_x f(\mu)+\frac{\epsilon^2}{2}\partial_{xx}f(\mu)+\mathcal{O}(\epsilon^3),\\
g(G_{x\pm\epsilon})&=g(G)\pm\epsilon\partial_x g(G)+\frac{\epsilon^2}{2}\partial_{xx}g(G)+\mathcal{O}(\epsilon^3),
\end{aligned}
\tag{S.25}
$$

where all terms on the right of (S.25) are evaluated at (x,t). Upon substituting (S.25) into (S.24), we obtain

$$
\begin{aligned}
&\rho_a(x,t+\tau|S|)-\rho_a(x,t)\\
&\quad=\Big(-\frac{\epsilon}{|S|}\rho_a(1-\rho_a-\rho_m)\partial_x(f(\mu)(1-2g(G)))\\
&\qquad-\frac{\epsilon}{|S|}(1-2\rho_a-\rho_m)f(\mu)(1-2g(G))\partial_x\rho_a\\
&\qquad+\frac{\epsilon}{|S|}\rho_a\partial_x f(\mu)\partial_x\rho_a+\frac{\epsilon}{|S|}\rho_a f(\mu)(1-2g(G))\partial_x\rho_m\\
&\qquad-\frac{\epsilon^2}{2|S|}\rho_a(1-\rho_a-\rho_m)\partial_{xx}f(\mu)\\
&\qquad+\frac{\epsilon^2}{2|S|}(1-\rho_m)f(\mu)\partial_{xx}\rho_a+\frac{\epsilon^2}{2|S|}\rho_a f(\mu)\partial_{xx}\rho_m\Big)+\mathcal{O}(\epsilon^3),
\end{aligned}
\tag{S.26}
$$

which can be rewritten as

$$\begin{aligned}
&\rho_a(x,t+\tau|S|)-\rho_a(x,t)\\
&\quad=\partial_x\Big(\frac{\epsilon^2}{2|S|}f(\mu)(1-\rho_a-\rho_m)\partial_x\rho_a+\frac{\epsilon^2}{2|S|}f(\mu)\rho_a\partial_x(\rho_a+\rho_m)\\
&\qquad-\frac{\epsilon^2}{2|S|}\rho_a(1-\rho_a-\rho_m)\partial_x f(\mu)-\frac{\epsilon}{|S|}f(\mu)(1-2g(G))\rho_a(1-\rho_a-\rho_m)\Big)\\
&\qquad+\mathcal{O}(\epsilon^3).
\end{aligned} \tag{S.27}$$

Dividing the resulting expression by $\tau|S|$ yields

$$\begin{aligned}
&\frac{\rho_a(x,t+\tau|S|)-\rho_a(x,t)}{\tau|S|}\\
&\quad=\partial_x\Big(\frac{\epsilon^2}{2\tau|S|^2}f(\mu)(1-\rho_a-\rho_m)\partial_x\rho_a+\frac{\epsilon^2}{2\tau|S|^2}f(\mu)\rho_a\partial_x(\rho_a+\rho_m)\\
&\qquad-\frac{\epsilon^2}{2\tau|S|^2}\rho_a(1-\rho_a-\rho_m)\partial_x f(\mu)\\
&\qquad-\frac{\epsilon}{\tau|S|^2}f(\mu)(1-2g(G))\rho_a(1-\rho_a-\rho_m)\Big).
\end{aligned} \tag{S.28}$$

We assume that the jump probability $f(\mu)$ is $\mathcal{O}(\tau/\epsilon^2)$ and the direction probability $1-2g(G)$ is $\mathcal{O}(\epsilon)$, such that the limits

$$\lim_{\epsilon,\tau\downarrow 0}\left(\frac{\epsilon^2 f(\mu)}{2\tau|S|}\right)=D_a(\mu),\quad \lim_{\epsilon,\tau\downarrow 0}\left(\frac{\epsilon f(\mu)(1-2g(G))}{\tau|S|}\right)=v_a(\mu,G) \tag{S.29}$$

exist, where $D_a(\mu)$ and $v_a(\mu,G)$ are dimensionless functions. The diffusion limit of (S.28) results in a nonlinear drift-diffusion equation

$$\begin{aligned}
\partial_t\rho_a=\partial_x\Big(&D_a(\mu)(1-\rho_a-\rho_m)\partial_x\rho_a+D_a(\mu)\rho_a\partial_x(\rho_a+\rho_m)\\
&-\rho_a(1-\rho_a-\rho_m)\partial_x D_a(\mu)-v_a(\mu,G)\rho_a(1-\rho_a-\rho_m)\Big).
\end{aligned} \tag{S.30}$$

The first term represents the diffusion of the A-cell subpopulation, where the factor $(1-\rho_a-\rho_m)$ represents the decrease of diffusivity due to the volume exclusion interaction of the total population. The last term corresponds to the drift flux of the A-cell subpopulation, where the factor $(1-\rho_a-\rho_m)$ represents the decrease of the drift velocity due to the exclusion of the total cell population.

3.2 Scaling limit of the combined phenotypic switch and cell migration process

In a next step, we take into account the phenotypic switch step dynamics. According to our model Rule (R1), a cell can change its phenotype first and then attempts to move. The global transition probabilities (S.17) then reads

$$P(\zeta,\nu):=\begin{cases}\frac{1}{|S|}W_1(r,r') & \text{if } \mathcal{R}^{\text{PS}}(\zeta(r))=\nu(r')=1,\zeta(r')=\nu(r)=0\\ \frac{1}{|S|}W_2(r,r') & \text{if } \mathcal{R}^{\text{PS}}(\zeta(r))=\nu(r')=2,\zeta(r')=\nu(r)=0\\ \frac{1}{|S|}\sum_{r\in S}W_1(r,r)+W_2(r,r) & \text{if } \zeta=\nu\\ 0 & \text{else,}\end{cases} \tag{S.31}$$

for $\zeta,\nu\in\mathcal{E}^S$ and $r'\in N_r$.

Accordingly, the discrete conservation equation (S.18) becomes

$$\begin{aligned}
P(\eta(r,k+1)=u)-&P(\mathcal{R}(\eta(r,k))=u)\\
&=\sum_{r'\in\mathcal{N}_r\backslash\{r\}}\frac{1}{|S|}W_u(r',r)P(\eta(r',k)=u,\mathcal{R}^{\text{PS}}(\eta(r,k))=0)\\
&\quad-\sum_{r'\in\mathcal{N}_r\backslash\{r\}}\frac{1}{|S|}W_u(r,r')P((\eta(r',k)=0,\mathcal{R}^{\text{PS}}(\eta(r,k))=u),
\end{aligned} \tag{S.32}$$

where $u \in \{1,2\}$. The phenotypic switch transition rule $\mathcal{R}(\eta(r,k))$ is applied prior to the actual migration process. The probability to be in state $u \in \mathcal{E}$ after application of the phenotypic switch rule is

$$P(\mathcal{R}(\eta(r,k)) = u) = \sum_{v \in \mathcal{E}} W(v,u) P(\eta(r,k) = v), \quad u \in \mathcal{E}, \tag{S.33}$$

where $W(u,v)$ specifies the phenotypic switch probability from state u to v given in (S.2).

Let us consider the change in mean A-cell density. Inserting (S.33) into (S.32), and invoking the mean-field assumption, we obtain

$$\begin{aligned}
&\rho_a(r,k) - \Big(W(0,1)(1-\rho_a(r,k)-\rho_m(r,k)) + W(1,1)\rho_a(r,k) + W(2,1)\rho_m(r,k)\Big) \\
&= \sum_{r' \in \mathcal{N}_r \setminus \{r\}} \frac{1}{|S|} W_1(r',r)\rho_a(r',k)\Big(W(0,0)(1-\rho_a(r,k)-\rho_m(r,k)) \\
&\qquad + W(1,0)\rho_a(r,k) + W(2,0)\rho_m(r,k)\Big) \\
&\quad - \sum_{r' \in \mathcal{N}_r \setminus \{r\}} \frac{1}{|S|} W_1(r,r')(1-\rho_a(r',k)-\rho_m(r',k))\Big(W(0,1)(1-\rho_a(r,k)-\rho_m(r,k)) \\
&\qquad + W(1,1)\rho_a(r,k) + W(2,1)\rho_m(r,k)\Big).
\end{aligned} \tag{S.34}$$

Using (S.2), the definition of the phenotypic switch probability W, (S.34) becomes

$$\begin{aligned}
&\rho_a(r,k) - (1-p_\alpha\mu(r,k))\rho_a(r,k) + p_\beta(1-\mu(r,k))\rho_m(r,k) \\
&= \sum_{r' \in \mathcal{N}_r \setminus \{r\}} \frac{1}{|S|} W(r',r)\rho_a(r',k)(1-\rho_a(r,k)-\rho_m(r,k)) \\
&\quad - \sum_{r' \in \mathcal{N}_r \setminus \{r\}} \frac{1}{|S|} W(r,r')(1-\rho_a(r',k)-\rho_m(r',k))\Big((1-p_\alpha\mu(r,k))\rho_a(r,k) \\
&\qquad + p_\beta(1-\mu(r,k))\rho_m(r,k)\Big).
\end{aligned} \tag{S.35}$$

In the next steps, we consider space and time scaling, expand terms in a Taylor series around x, divide by $\tau|S|$ and take the diffusive limit. Taking into account the limit behavior of $p_\alpha(\tau) = \alpha\tau + \mathrm{o}(\tau)$ and $p_\beta(\tau) = \beta\tau + \mathrm{o}(\tau)$, we finally obtain

$$\begin{aligned}
\partial_t \rho_a = \partial_x \Big(&D_a(\mu)(1-\rho_a-\rho_m)\partial_x\rho_a + D_a(\mu)\rho_a\partial_x(\rho_a+\rho_m) \\
&- \rho_a(1-\rho_a-\rho_m)\partial_x D_a(\mu) - v_a(\mu,G)\rho_a(1-\rho_a-\rho_m)\Big) \\
&- \alpha\mu\rho_a + \beta(1-\mu)\rho_m,
\end{aligned} \tag{S.36}$$

where

$$D_a(\mu) = \lim_{\epsilon,\tau\downarrow 0}\left(\frac{\epsilon^2 f(\mu)}{2\tau|S|}\right), \quad v_a(\mu,G) = \lim_{\epsilon,\tau\downarrow 0}\left(\frac{\epsilon f(\mu)(1-2g(G))}{\tau|S|}\right). \tag{S.37}$$

For brevity, all terms in the above expressions are evaluated at (x,t).

3.3 Scaling limit of the ECM degradation

In our model, M-cells locally degrade ECM. In particular, an M-cell at node r degrades ECM with constant factor c_δ, see Rule (R2). Without the condition that only M-cells locally degrade ECM, the ECM degradation step would be purely deterministic, in particular, $\mu(r,k+1) = (1-c_\delta)\mu(r,k)$ for $\mu \in \mathcal{E}^S_{\text{ECM}}$. Including the M-cell condition, the probability for a node r to be in a specific state $u \in \mathcal{E}_{\text{ECM}}$ is given by

$$P(\mu(r,k+1) = u) = P\left(\mu(r,k) = \frac{1}{(1-c_\delta)}u, \eta(r,k) = 2\right). \tag{S.38}$$

Let $\rho_\mu(r,k) := \mathrm{E}(\mu(r,k))$ denote the mean ECM resistance density. By invoking the mean-field assumption, the change in ECM resistance density at node r during the next time step is then given by

$$\rho_\mu(r,k+1) - \rho_\mu(r,k) = -c_\delta\rho_\mu(r,k)P(\mathcal{R}^{\text{PS}}(\eta(r,k)) = 2). \tag{S.39}$$

Again, as in Section 3.1, we set $x = r\epsilon$ and $t = k\tau|S|$, $\epsilon, \tau \in \mathbb{R}_+$, and reinterpret $\rho_\mu(x,t)$ as continuous variable. We obtain

$$\partial_t \rho_\mu = -\delta \rho_\mu \rho_m, \tag{S.40}$$

where δ is the rate of ECM degradation. For brevity, all terms in equation (S.40) are evaluated at (x,t).

3.4 Analysis of the complete macroscopic model

The complete macroscopic description of our automaton dynamics is given by a system of coupled nonlinear partial differential equations (PDEs)

$$\begin{aligned}
\partial_t \rho_a = \partial_x \Big(& D_a(\mu)(1-\rho_a-\rho_m)\partial_x \rho_a + D_a(\mu)\rho_a \partial_x(\rho_a+\rho_m) \\
& - \rho_a(1-\rho_a-\rho_m)\partial_x D_a(\mu) - v_a(\mu, G)\rho_a(1-\rho_a-\rho_m) \Big) \\
& - \alpha\mu\rho_a + \beta(1-\mu)\rho_m \qquad \text{(S.41)} \\
\partial_t \rho_m = \partial_x \Big(& D_m(\mu)(1-\rho_a-\rho_m)\partial_x \rho_m + D_m(\mu)\rho_m \partial_x(\rho_a+\rho_m) \\
& - \rho_m(1-\rho_a-\rho_m)\partial_x D_m(\mu) - v_m(\mu, G)\rho_m(1-\rho_a-\rho_m) \Big) \\
& + \alpha\mu\rho_a - \beta(1-\mu)\rho_m \qquad \text{(S.42)} \\
\partial_t \rho_\mu = & -\delta\rho_\mu\rho_m. \qquad \text{(S.43)}
\end{aligned}$$

The above derived PDE system (S.41)-(S.43) is of interest in two respects. First, the numerical solution of the PDE system may provide a computational more efficient way to investigate the cell population migration distance d compared to the cellular automaton simulations. The latter is limited by its computational cost since the automaton model requires many repeated simulations to account for stochastic fluctuations. Second, a rigorous analysis of the complete PDE system (existence and uniqueness of solutions, traveling wave analysis, etc.) may allow to estimate precise parameter values and environmental conditions under which plastic migration behavior is beneficial for tumor invasion. The particular challenge of studying the derived PDE system lies in the analysis of the nonlinear drift-diffusion equations (S.41) and (S.42). The behavior of solutions of fully nonlinear systems like (S.41)-(S.42) are not known.

References

[1] Liggett TM. Stochastic Interacting Systems: Contact, Voter and Exclusion Processes. Springer, New York. 1999.

[2] Simpson MJ, Merrifield A, Landman KA, Hughes BD. Simulating invasion with cellular automata: connecting cell-scale and population-scale properties. Physical Review E 2007; 76: 021918.

[3] Simpson M, Landmann K, Hughes B. Multi-species simple exclusion processes. Phys. A 2009; 388:399-406.

[4] Schönfisch B. Simple individual based models of movement, alignment and schooling behaviour. Future Gen Comp Sys 2001; 17: 873-882.

4 Figures

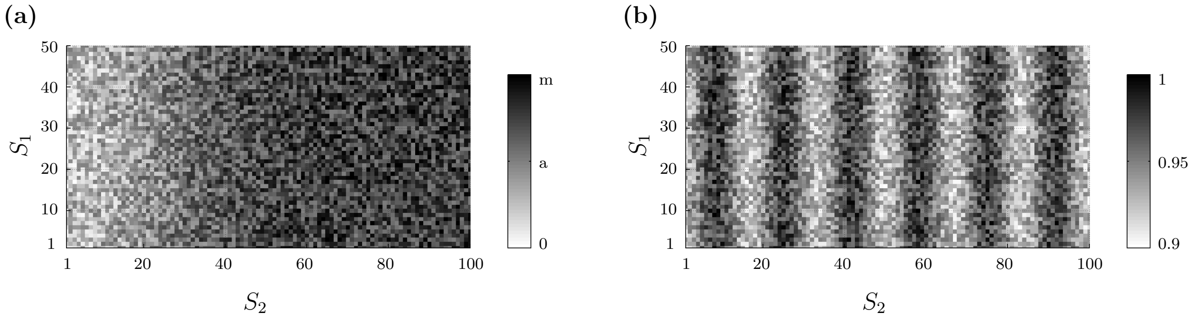

Figure S1: Initial ECM resistance distribution of differently structured heterogeneous ECM. (a) Heterogeneous ECM resistance distribution, defined by (a) equation (S.15), and (b) equation (S.16), with heterogeneity parameter $\theta = 0.5$.

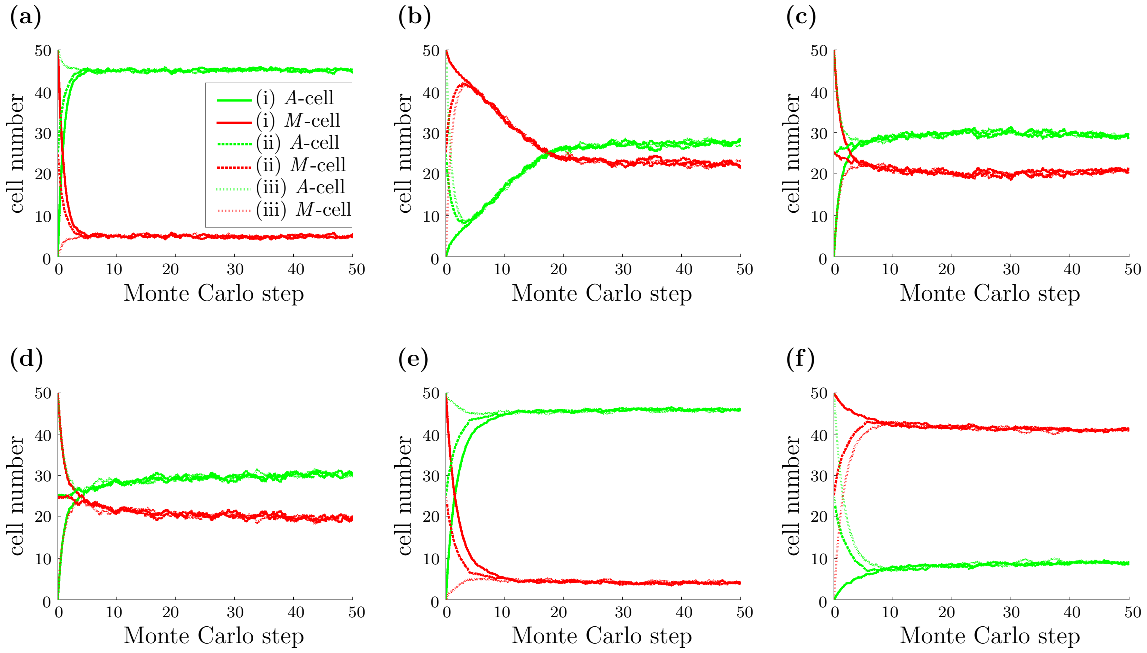

Figure S2: Equilibrium cell compositions depend on ECM structure and migration rate ratio but are always reached already after a few Monte Carlo steps, independent from the initial cell compositions. The figure shows the evolution of A- and M-cell number with different initial M-cell fractions: (i) $\gamma = 1$, (ii) $\gamma = 0.5$ and (iii) $\gamma = 0$, where the A-cell number is color indicated by green, M-cell number by red. Results are shown for different ECM conditions and for different α/β switch ratios: (a) homogeneous, low ECM resistance distribution with $\alpha/\beta = 1$; (b) homogeneous, high ECM resistance distribution with $\alpha/\beta = 1$; (c) heterogeneous ECM resistance, large migration rate ratio with $\alpha/\beta = 1$; (d) heterogeneous ECM resistance, small migration rate ratio with $\alpha/\beta = 1$; (e) heterogeneous ECM resistance, small migration rate ratio with $\alpha/\beta = 0.1$; (f) heterogeneous ECM resistance, small migration rate ratio with $\alpha/\beta = 10$.

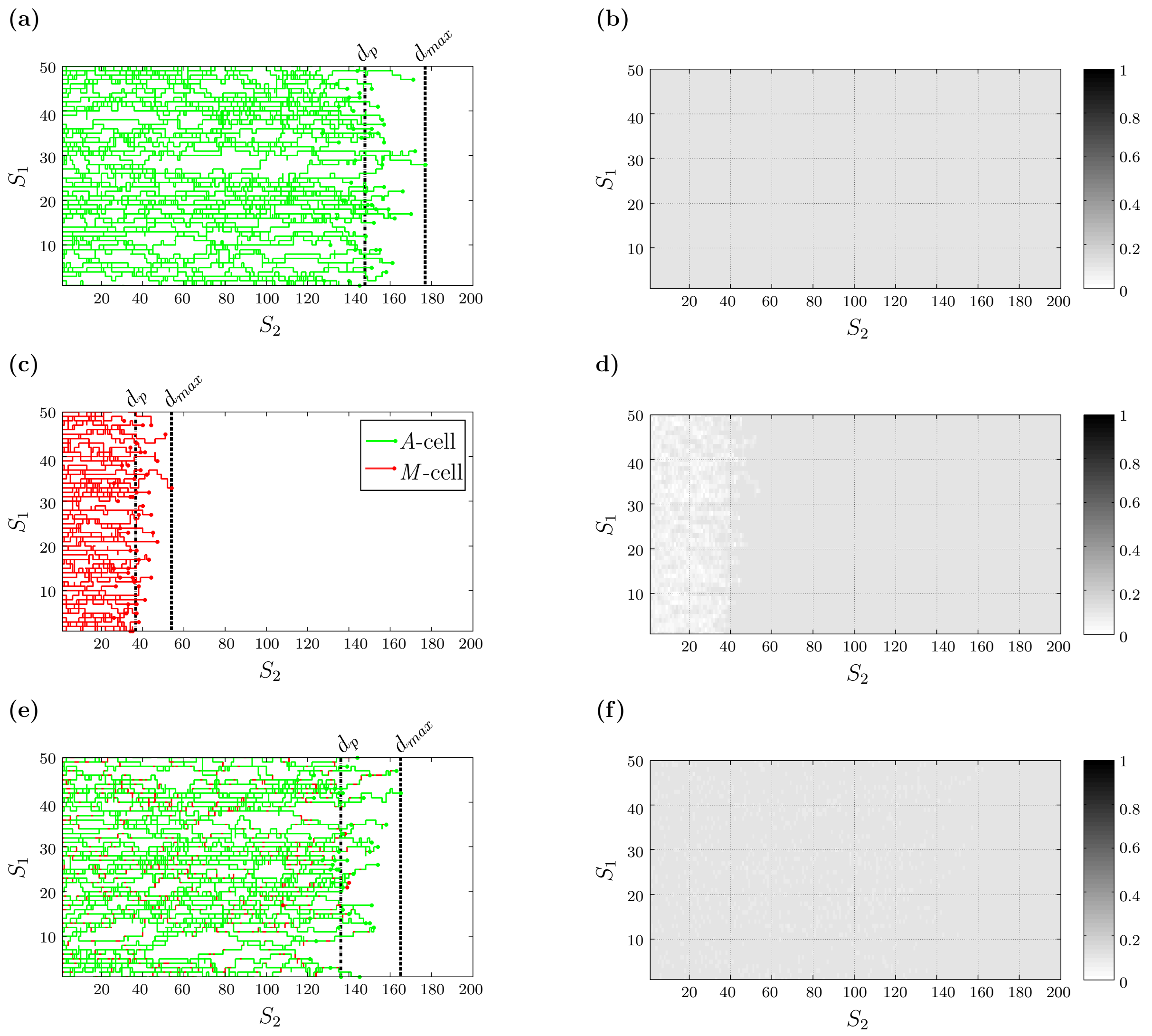

Figure S3: Individual cell trajectories under homogeneous, low ECM condition. Individual cell trajectories (a,c,e) are visualized together with the ECM resistance distribution (b,d,f) after 200 Monte Carlo steps. A-cells and their trajectory are color indicated by green, M-cells by red. (a) non-switching cell population with $\gamma = 0$ (pure A-cell population) for which (b) the ECM after the cell migration has not being changed. (c) non-switching cell population with $\gamma = 1$ (pure M-cell population) with (d) corresponding ECM after cell migration. (e) switching cell population with $\alpha/\beta = 1$ and (f) corresponding ECM. The initial ECM resistance distribution is modeled by $mu(r_1, r_2) = 0.1$. Simulation parameters are $c_M/c_A = 0.25$, $\kappa = 0.01$, $\delta = 0.1$.

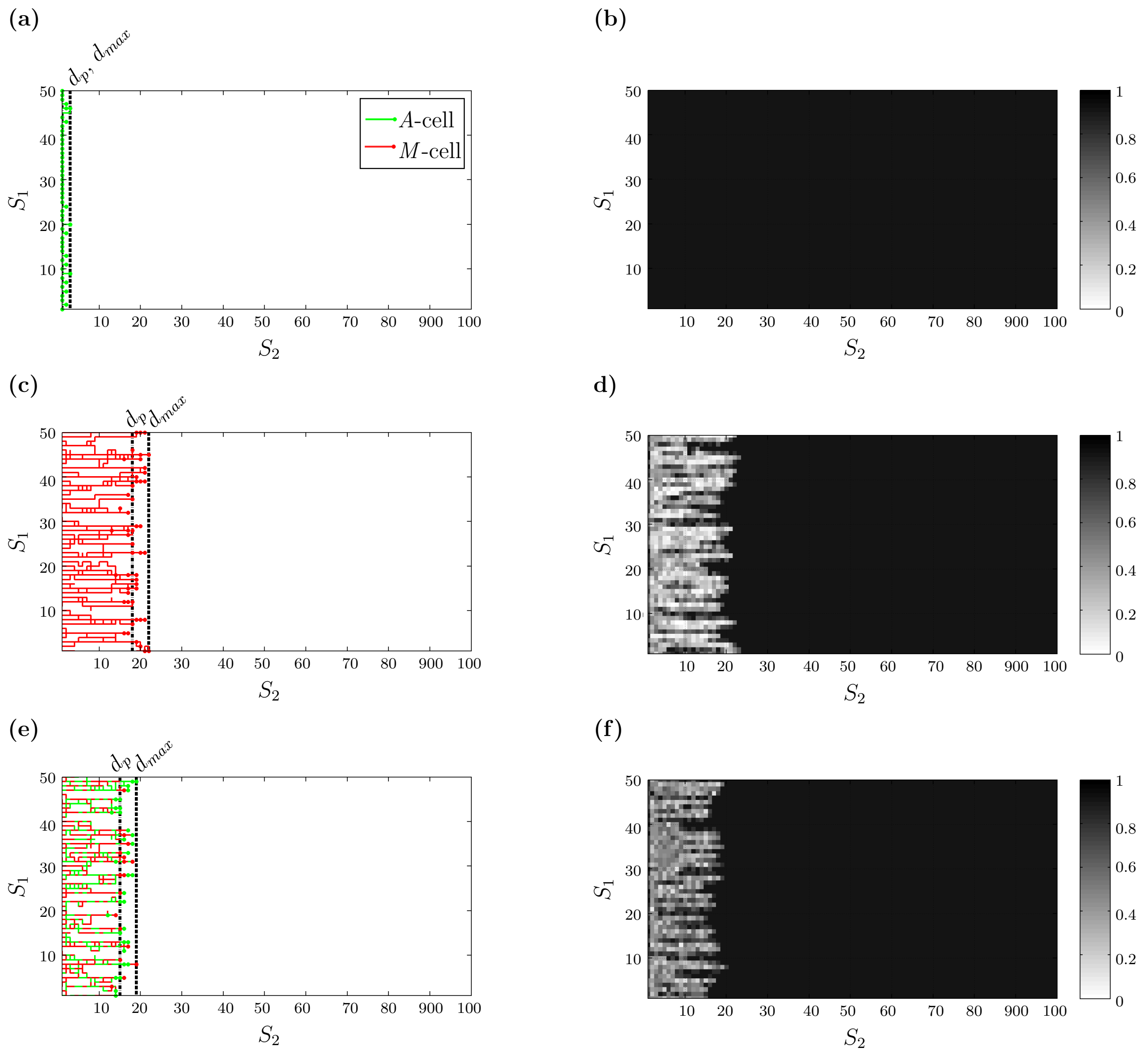

Figure S4: Individual cell trajectories under homogeneous, low ECM condition. Individual cell trajectories (a,c,e) are visualized together with the ECM resistance distribution (b,d,f) after 200 Monte Carlo steps. A-cells and their trajectory are color indicated by green, M-cells by red. (a) non-switching cell population with $\gamma = 0$ (pure A-cell population) for which (b) the ECM after the cell migration has not being changed. (c) non-switching cell population with $\gamma = 1$ (pure M-cell population) with (d) corresponding ECM after cell migration. (e) switching cell population with $\alpha/\beta = 1$ and (f) corresponding ECM. The initial ECM resistance distribution is modeled by $mu(r_1, r_2) = 0.9$. Simulation parameters are $c_M/c_A = 0.25$, $\kappa = 0.01$, $\delta = 0.1$.

(a) homogeneous, low ECM resistance

(b) homogeneous, high ECM resistance

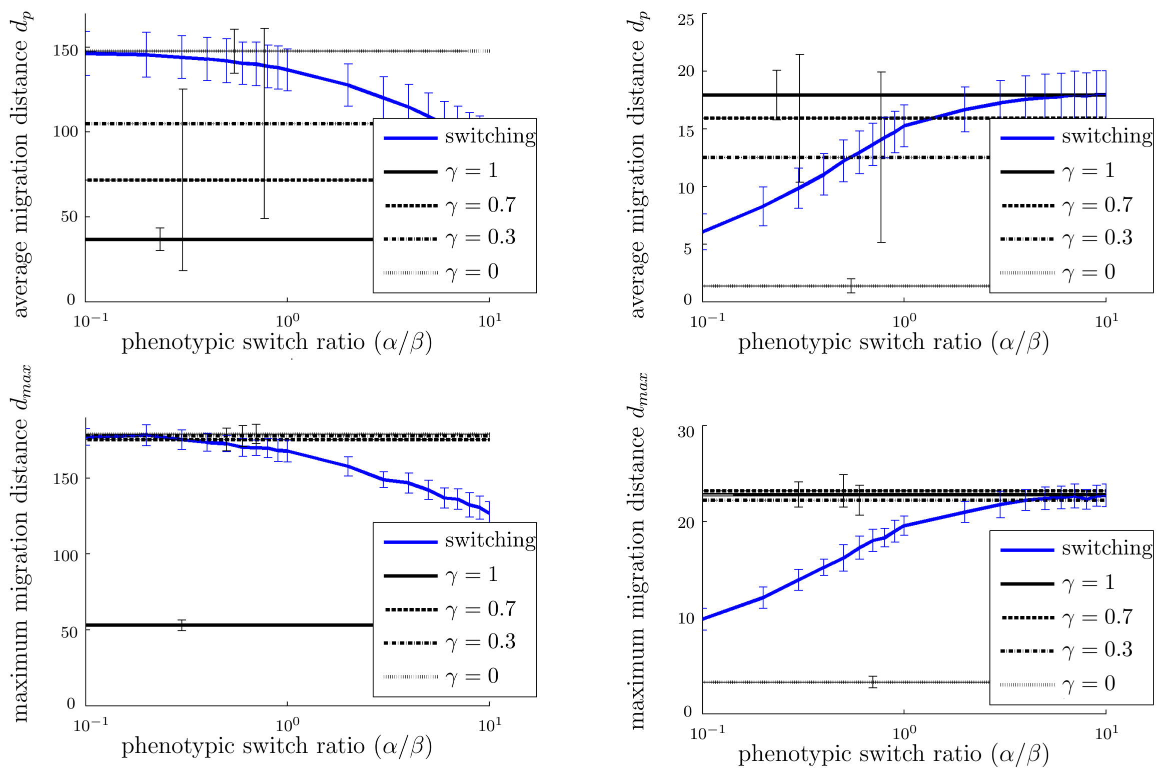

Figure S5: Advantage of non-switching behavior under homogeneous ECM conditions. The figure shows the average (top row) and maximum (bottom row) migration distance of switching and non-switching populations depending on the switch ratio α/β for different homogeneous ECM conditions: (a) homogeneous, low ECM resistance modeled by $\mu(r_1, r_2) = 0.1$ and (b) homogeneous, high ECM resistance with $\mu(r_1, r_2) = 0.9$. The blue line represents the switching populations, the black lines display non-switching populations with different M-cell fraction $\gamma \in \{0, 0.3, 0.7, 1\}$. Each simulation is run with 50 cells. Simulations are evaluated after 200 Monte Carlo steps, averaged over 50 independent simulations. The errorbars show the standard deviation of the average migration distance d_p within the simulations (top row) and the standard deviation of the maximum migration distance d_{max} between the simulations (bottom row). Simulation parameters are $c_M/c_A = 0.25$, $\kappa = 1$, $\delta = 0.1$.

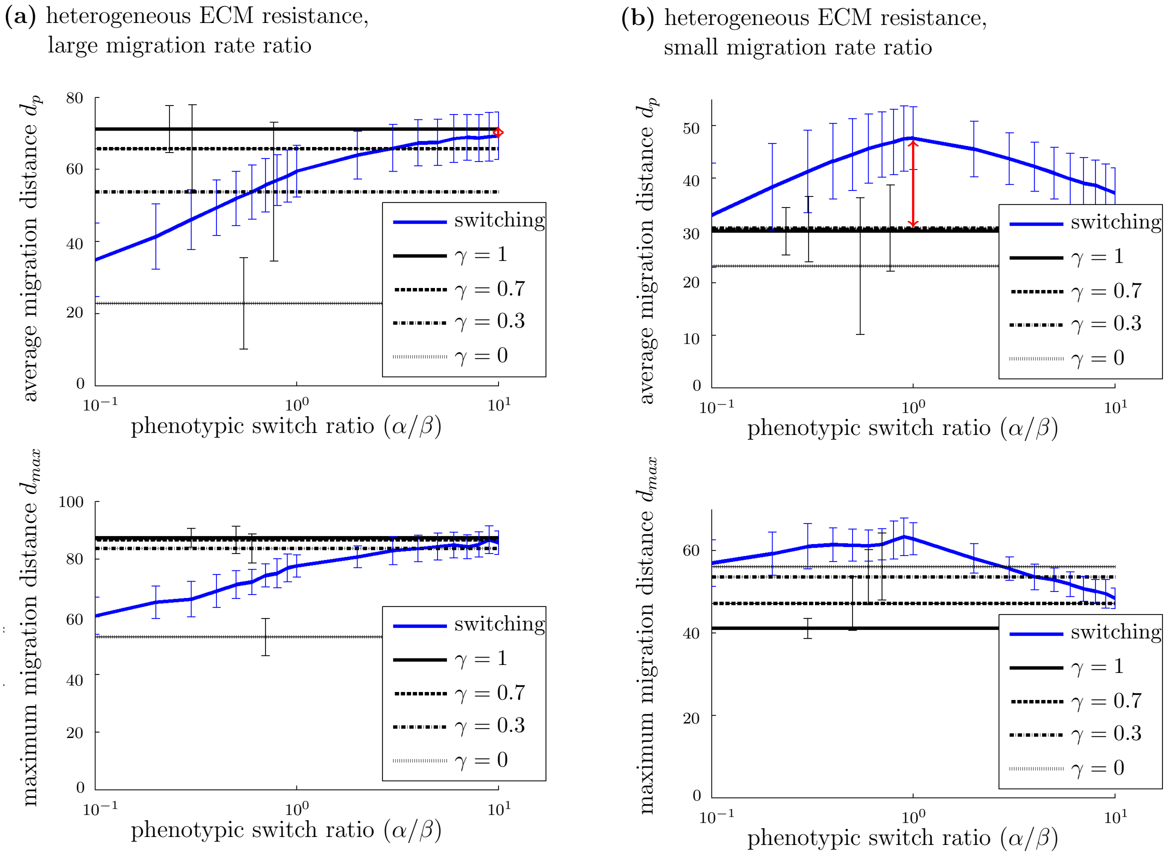

Figure S6: Migration plasticity can be advantageous under heterogeneous, highly structured ECM conditions. The figure shows the average (top row) and maximum (bottom row) migration distance d_p of switching and non-switching populations depending on the switch ratio α/β under heterogeneous ECM condition (modeled by equation (2) in the main text with $\theta = 0.5$) and with different migration rate ratios: (a) $c_M/c_A = 0.75$ and (b) $c_M/c_A = 0.25$. The blue line represents the switching populations, the black lines display non-switching populations with different M-cell fraction $\gamma \in \{0, 0.3, 0.7, 1\}$. Each simulation is run with 50 cells. Simulations are evaluated after 200 Monte Carlo steps, averaged over 50 independent simulations. The errorbars show the standard deviation of the average migration distance d_p within the simulations (top row) and the standard deviation of the maximum migration distance d_{max} between the simulations (bottom row). Simulation parameters are $\kappa = 1$, $\delta = 0.1$. The red arrow indicates the difference Δd_p between the switching population with maximum average migration distance d_p with respect to varied α/β ratio and the non-switching population with maximum d_p with respect to varied γ fraction, which is the observable analyzed in the Figure 7 of the main text.

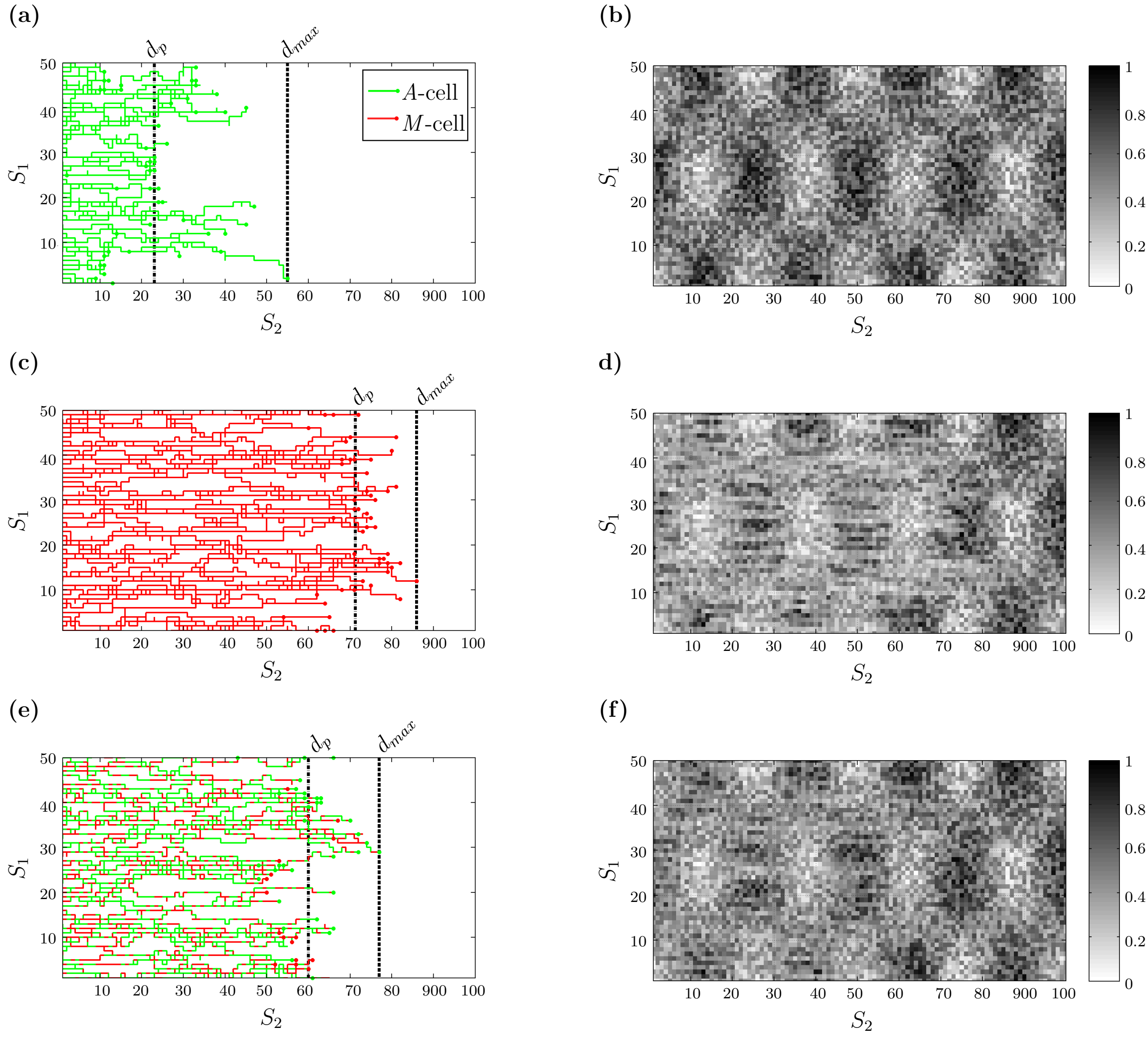

Figure S7: Individual cell trajectories under heterogeneous ECM conditions with large migration rate ratio. Individual cell trajectories (a,c,e) are visualized together with the ECM resistance distribution (b,d,f) after 200 Monte Carlo steps. A-cells and their trajectory are color indicated by green, M-cells by red. (a) non-switching cell population with $\gamma = 0$ (pure A-cell population) for which (b) the ECM after the cell migration has not being changed. (c) non-switching cell population with $\gamma = 1$ (pure M-cell population) with (d) corresponding ECM after cell migration. (e) switching cell population with $\alpha/\beta = 1$ and (f) corresponding ECM. The vertical dashed lines in (a), (c) and (e) mark the average distance d_p, the dotted line specifies the maximum migration distance d_{max}. The red arrow indicates the difference Δd_p between the maximum average migration distance d_p of the switching population and the non-switching population with $\gamma = 0$. The initial ECM resistance distribution is modeled by equation (2) in the main text with $\theta = 0.5$. Simulation parameters are $c_M/c_A = 0.75$, $\kappa = 0.01$, $\delta = 0.1$.

(a) homogeneous, low ECM resistance **(b)** homogeneous, high ECM resistance

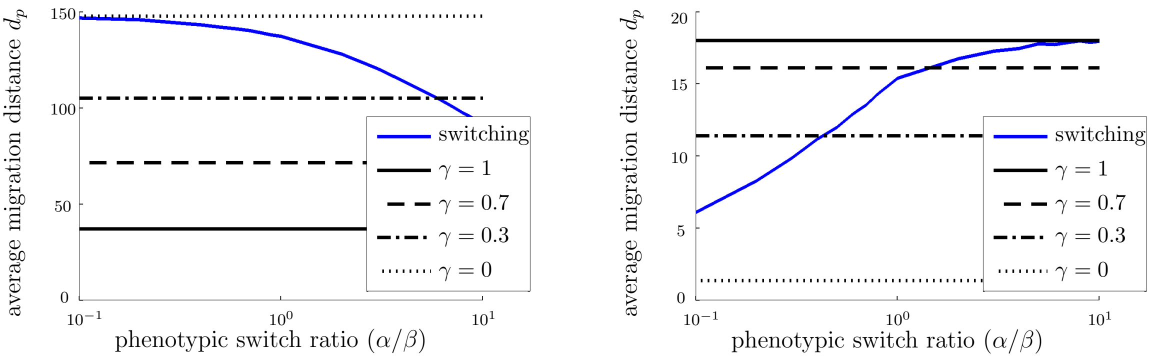

Figure S8: Advantage of non-switching behavior under homogeneous ECM conditions is independent of chemotactic gradient sensitivity. The figure shows the average migration distance d_p of switching and non-switching populations depending on the switch ratio α/β, for low chemotactic responsiveness ($\kappa = 0.01$) and under different homogeneous ECM conditions: (a) homogeneous, low ECM resistance modeled by $\mu = 0.1$ and (b) homogeneous, high ECM resistance with $\mu = 0.9$. The blue line represents the switching populations, the black lines display non-switching populations with different M-cell fraction $\gamma \in \{0, 0.3, 0.7, 1\}$. Each simulation is run with 50 cells. Simulations are evaluated after 200 Monte Carlo steps, averaged over 50 independent simulations. The standard deviation is not shown as it is negligible. Simulation parameters are $c_M/c_A = 0.25$, $\delta = 0.1$.

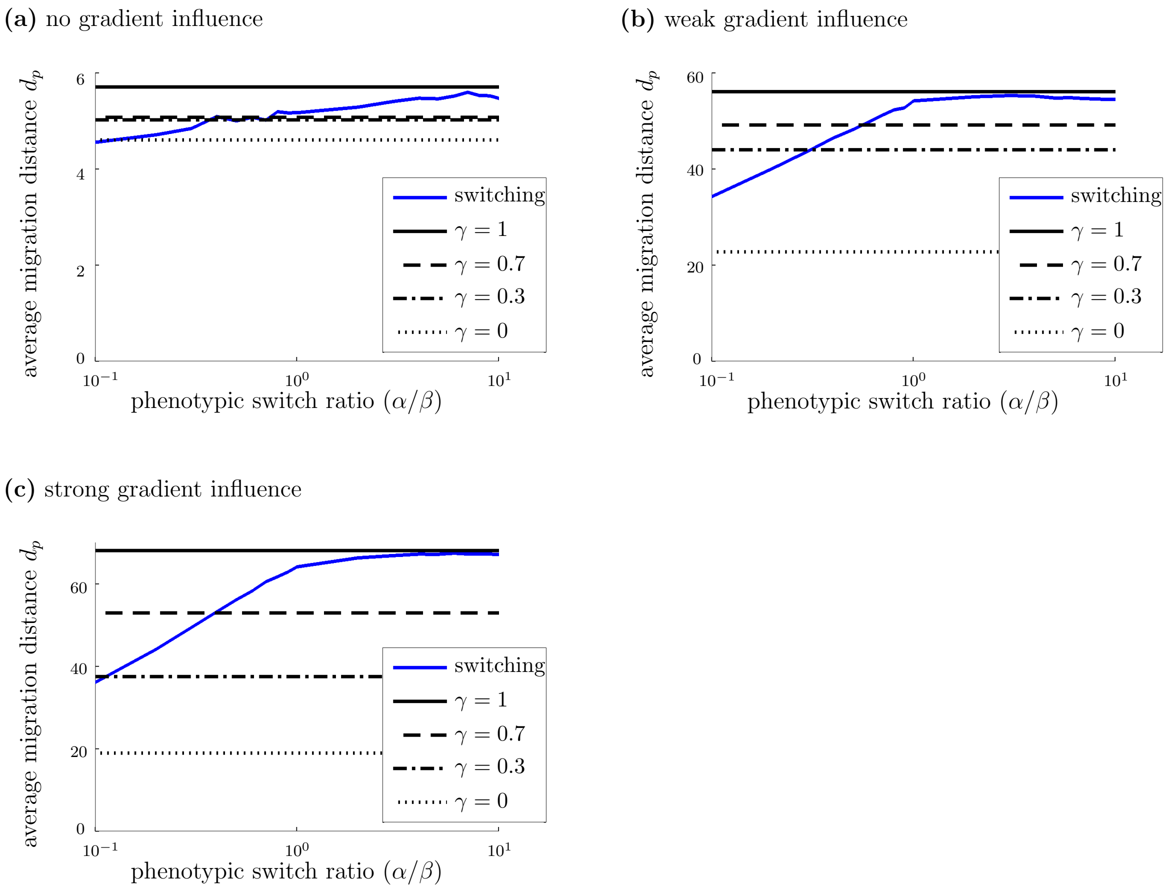

Figure S9: Advantage of non-switching behavior under heterogeneous, weakly structured ECM and low chemotactic responsiveness. The figure shows the average migration distance d_p of switching and non-switching populations depending on the switch ratio α/β under heterogeneous ECM condition (modeled by equation (2) in the main text, with heterogeneity parameter $\theta = 0.5$) and with different chemotactic responsiveness: (a) no gradient responsiveness modeled by $\kappa = 0$, (b) low gradient responsiveness modeled by $\kappa = 0.01$ and (c) high gradient responsiveness $\kappa = 1$. The blue line represents the switching populations, the black lines display non-switching populations with different M-cell fraction $\gamma \in \{0, 0.3, 0.7, 1\}$. Each simulation is run with 50 cells. Simulations are evaluated after 200 Monte Carlo steps, averaged over 50 independent simulations. The standard deviation is not shown as it is negligible. Simulation parameters are $c_M/c_A = 0.5$, $\delta = 0.1$.

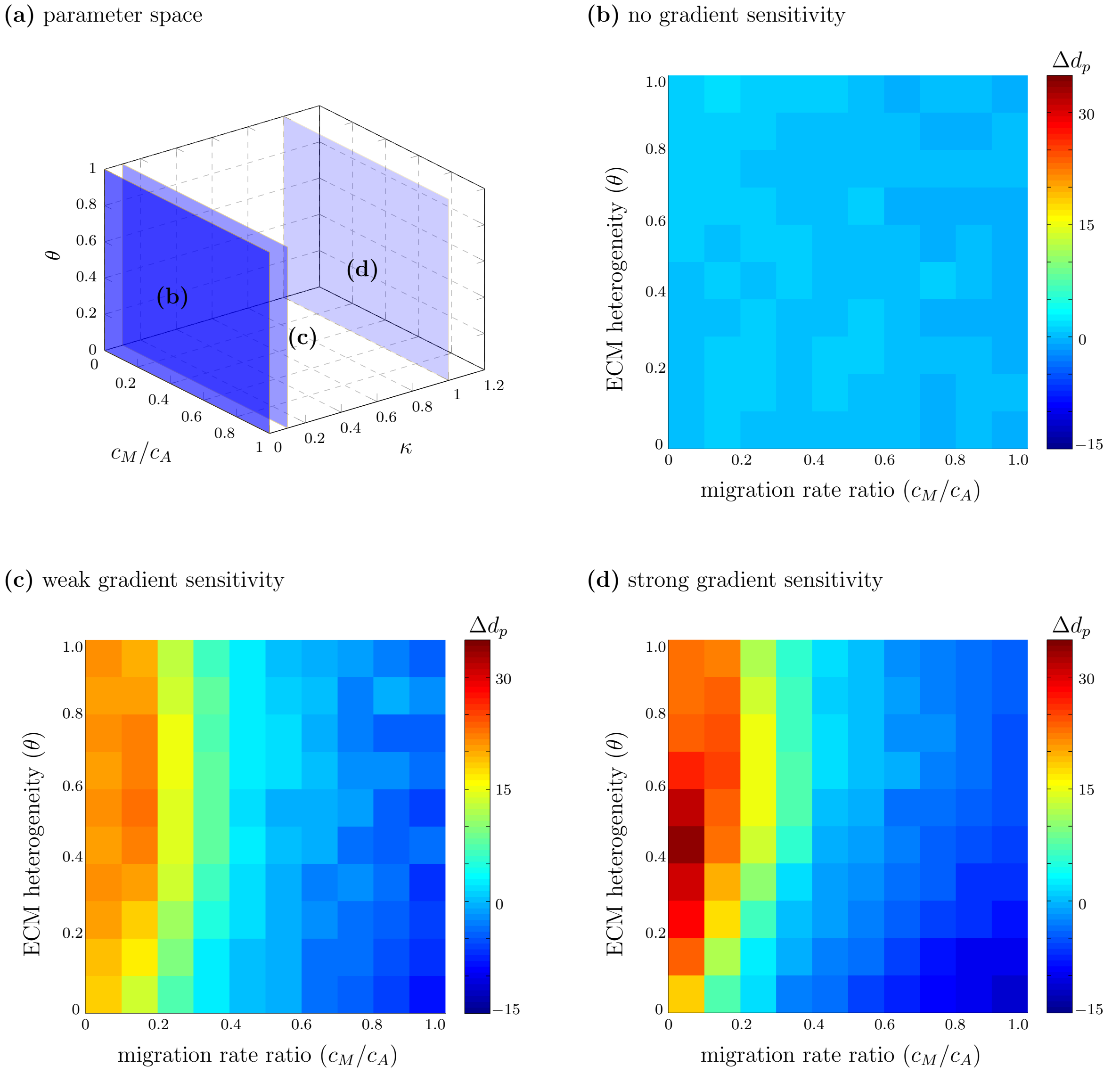

Figure S10: The difference Δd_p between the maximum average migration distance d_p of switching and non-switching populations depends on the ECM heterogeneity (θ) and the migration rate ratio (c_M/c_A). (a) The parameter space is defined by $\theta \in [0,1]$, $\kappa \in [0,1]$ and $c_M/c_A \in [0,1]$. For each point in the parameter space, we measure the average migration distance d_p of switching and non-switching populations as described in the main text. The three different blue shaded surfaces mark the $(\theta, c_M/c_A)$-planes analyzed in (b)-(d). (b)-(d) Difference Δd_p between the switching population with maximum average migration distance d_p with respect to varied switch ratio α/β and the non-switching population with maximum average migration distance d_p with respect to varied γ fraction. The phase diagram are shown for different chemotactic responsiveness: (b) without chemotactic responsiveness ($\kappa = 0$); (c) a low chemotactic responsiveness ($\kappa = 0.01$); (d) a high chemotactic responsiveness ($\kappa = 1$). Red to turquois areas in the phase diagram indicate parameter combinations $(\theta, c_M/c_A)$ for which the maximum migration distance d_p of the switching behavior is highest, whereas blue refers to an advantage of a non-switching behavior.
